# The Role of Ferroptosis in Women's Health and Diseases

**DOI:** 10.1002/mco2.70296

**Published:** 2025-08-15

**Authors:** Qiang Xu, Chongying Zhu, Lin Li, Jiayong Li, Zihao An, Chao Tang

**Affiliations:** ^1^ National Clinical Research Center for Child Health of the Children's Hospital Zhejiang University School of Medicine Hangzhou China; ^2^ The Department of Obstetrics and Gynecology Ruijin Hospital, Shanghai Jiaotong University School of Medicine Shanghai China; ^3^ Department of Urology Third Affiliated Hospital of Naval Medical University Shanghai China; ^4^ State Key Laboratory of Ophthalmology Zhongshan Ophthalmic Center Sun Yat‐sen University Guangzhou China

**Keywords:** ferroptosis, gynecological diseases, mechanism, obstetric diseases, targeted therapy

## Abstract

Currently, there are many diseases worldwide that seriously affect women's health. Among these, gestational disorders and female cancers such as ovarian cancer are particularly notable for their high morbidity and mortality rates. Over the past decade, ferroptosis, a distinct form of programmed cell death primarily driven by iron‐dependent phospholipid peroxidation, has been implicated in the pathogenesis of various female‐related diseases. Despite many studies individually reporting that ferroptosis plays a crucial pathogenic role in common female disorders, there has yet to be a systematic overview addressing the mechanisms linking ferroptosis with women's health and disease. Thus, we herein provide a comprehensive review of the relationship between cellular pathways of ferroptosis and women's health and disease and describe the current progress of targeted therapy for ferroptosis. Following a succinct introduction to the disease, we summarize the regulatory role of ferroptosis in women's health and its implications for disease progression, with the aim of facilitating a clearer understanding of the relationship between ferroptosis and women's health. Finally, we discuss the emerging challenges and opportunities presented by various agonists or inhibitors targeting ferroptosis as potential therapeutic strategies for female‐related diseases, providing additional protective approaches contributing to female health.

## Graphical abstract

At present, there are many common women's diseases, such as preeclampsia (PE), ovarian cancer, and cervical cancer, endangering women's health. These diseases all over the world are characterized by high morbidity and mortality. In the past decade, ferroptosis, a unique pattern of programmed cell death primarily driven by iron‐dependent phospholipid peroxidation, has been associated with the development of various diseases and response to therapy. In recent years, numerous studies have provided convincing evidence that ferroptosis plays a key pathogenic role in common obstetric and gynecological diseases. In this review, we detail our current knowledge on the major pathways of ferroptosis, summarizing their regulation during the progression of female reproductive diseases. Finally, we discuss new challenges and opportunities for using ferroptosis as a potential strategy for the treatment of obstetric‐related diseases.

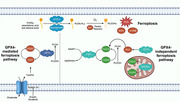

## 1 Introduction

In 2012, the concept of ferroptosis was first introduced in the study by Dixon et al. [1]. Different from other programmed cell death methods such as apoptosis and necrosis, the morphological characteristics of ferroptosis are mitochondrial atrophy, increased bilayer membrane density, and decreased mitochondrial cristae, but there is no rupture of the cell membrane and no chromatin condensation [2, 3].

Recent studies have indicated that ferroptosis may be involved in regulating the pathophysiological processes of various female reproductive disorders, including trophoblast invasion, oocyte development, embryonic development, endometrial shedding, and oxidative stress and metabolism of granulation cells [4]. In addition, ferroptosis also plays a significant role in the development of common gynecologic tumors (such as ovarian cancer, endometrial cancer, cervical cancer, etc.) [5, 6, 7, 8]. It is not difficult to conclude that ferroptosis is closely related to the field of female health and disease and is an emerging focus for future research in women.

At present, there have been reports summarizing the correlations between other systems and ferroptosis [9, 10, 11, 12]. Although there are many reports on the relationship between ferroptosis and women's health and disease, systematic descriptions are lacking [13, 14]. This review briefly describes the pathogenesis of common female diseases, generalizes the signaling mechanisms of ferroptosis in female health and disease, and finally summarizes the function and significance of ferroptosis in various female diseases, indicating ferroptosis may be a possible approach for treating various female diseases.

## 2 Molecular Mechanism of Ferroptosis

### 2.1 The Characteristics of Ferroptosis and Metabolic Dysregulation

Ferroptosis is usually accompanied by iron overload and accumulation of phospholipid hydroperoxides (PLOOHs) [15, 16], and plays a key role in ferroptosis‐induced programmed death as the initiator of downstream cascades [17]. Polyunsaturated fatty acids (PUFAs), especially arachidonic acid and adrenal acid, are lipoylated by acyl‐CoA synthetase long‐chain family member 4 (ACSL4) and lipoxygenase through the lipoxygenase pathway [18, 19, 20], and are subsequently fixed on the membrane phospholipids to form polyunsaturated fatty acids (PUFA‐PLs) [21]. Free Fe^2+^ can catalyze membrane high expression of PUFA‐PLs with molecular oxygen in the Fenton reaction, electron transfer to hydrogen peroxide and oxygen free radicals, leading to reactive oxygen species and decomposition products of lipid peroxides such as 4‐hydroxynonenal (4‐HNE) and malondialdehyde (MDA) accumulation [22, 23]. Ferroptosis is the result of the imbalance of the cellular redox system and lipid oxide metabolism disorders, which leads to the impairment of the intracellular antioxidant clearance mechanism through a variety of ways, and the accumulation of specific lipid ROS, resulting in irreparable damage to the cell membrane and further cell death [24, 25].

### 2.2 The Key Regulatory Molecule of Ferroptosis

Ferroptosis is divided into classical signaling pathways and nonclassical signaling pathways, and there are many key regulatory molecules in these processes, including GPX4, System Xc‐, ACSL4, etc. [18, 19, 20]. We herein elaborate on the role and significance of each regulatory molecule by describing the mechanism.

#### 2.2.1 Classical Glutathione Peroxidase 4 (GPX4)‐Mediated Ferroptosis Pathway

The typical regulatory pathways are the exogenous pathway with the transporter as the core and the endogenous pathway mediated by GPX4 [26]. Ferroptosis is characterized by impairment of intracellular antioxidant defenses, accompanied by decreased glutathione cycling and decreased activity of the core antioxidant enzyme GPX4. The cystine/glutamate antiporter system (System Xc) is distributed in the cell membrane. As the main subunit of System Xc, Solute carrier protein 7 family member 11(SLC7A11) mediates the exchange of glutamate and cystine and provides an active substrate for the synthesis of reduced glutathione (GSH) [27]. GPX4 reduces phospholipid peroxide PLOOHs by consuming GSH, inhibits the activation of arachidonic acid metabolic enzymes in microsomal lipid peroxidation and phospholipid peroxidation pathway, and is the only antioxidant enzyme that directly clears phospholipid peroxides in cells [28]. When GSH is in short supply, it directly affects the function of GPX4 [29]. Cysteine, glutamine metabolism, and some small molecule compounds, such as Erastin and Sorafenib, lead to the depletion of intracellular GSH cycle, the accumulation of oxidative GSH, and the inactivation of GPX4 by inhibiting the activity of the cell membrane transport carrier System Xc, and then increase the toxicity of lipid hydroperoxides and trigger ferroptosis [29, 30, 31].

#### 2.2.2 GPX4‐Independent Ferroptosis Pathway

Recently, the tetrahydrobiopterin (BH4) pathway has been reported as a compensatory mechanism for the *GPX4*‐deficient enzyme catalytic system [32]. Kraft et al. reported that BH4 can inhibit ferroptosis by producing CoQ10 or blocking the production of specific lipid hydroperoxide, and guanosine triphosphate cyclohydrolase 1 (GCH1) acts as the rate‐limiting step of BH4 production to dynamically regulate ferroptosis [33]. In particular, an antioxidant defense mechanism parallel to the mitochondrial GPX4 pathway is proposed [34]: dihydroorotate dehydrogenase (DHODH) is located on the inner mitochondrial membrane surface and catalyzes the generation and metabolism of pyrimidine nucleotides. DHODH acts enzymatically to oxidize dihydroorotate while transferring electrons to CoQ10 and reducing them to free radicals to capture the antioxidant CoQH2, regulating the sensitivity of cells to GPX4 inhibitors and further attenuating the mitochondrial lipid peroxidation and ferroptosis cascade mediated by GPX4 inactivation. The above outcome illustrates that DHODH plays a regulatory role as a background‐dependent antioxidant defense mechanism within the cell. In addition, studies have reported that ferroptosis suppressor protein 1 (FSP1) has an antiferroptosis effect after being modified by myristate [35, 36]. FSP1 is mainly localized in the cell membrane and lipid droplets, where it uses nicotinamide adenine dinucleotide phosphate (NADPH) to catalyze the production of mitochondrial electron transport chain coenzyme ubiquinone (CoQ10), In its reduced form, ubiquinol (CoQH2) traps lipid peroxy radicals and terminates the lipid peroxidation process [37, 38]. However, this ferroptosis inhibitor effect is independent of GSH level, GPX4 activity, or oxidizable fatty acid content, suggesting that the FSP1‐CoQ10‐NADPH pathway, as an independent and parallel pathway, plays an essential role in maintaining phospholipid redox homeostasis [36].

**FIGURE 1:**
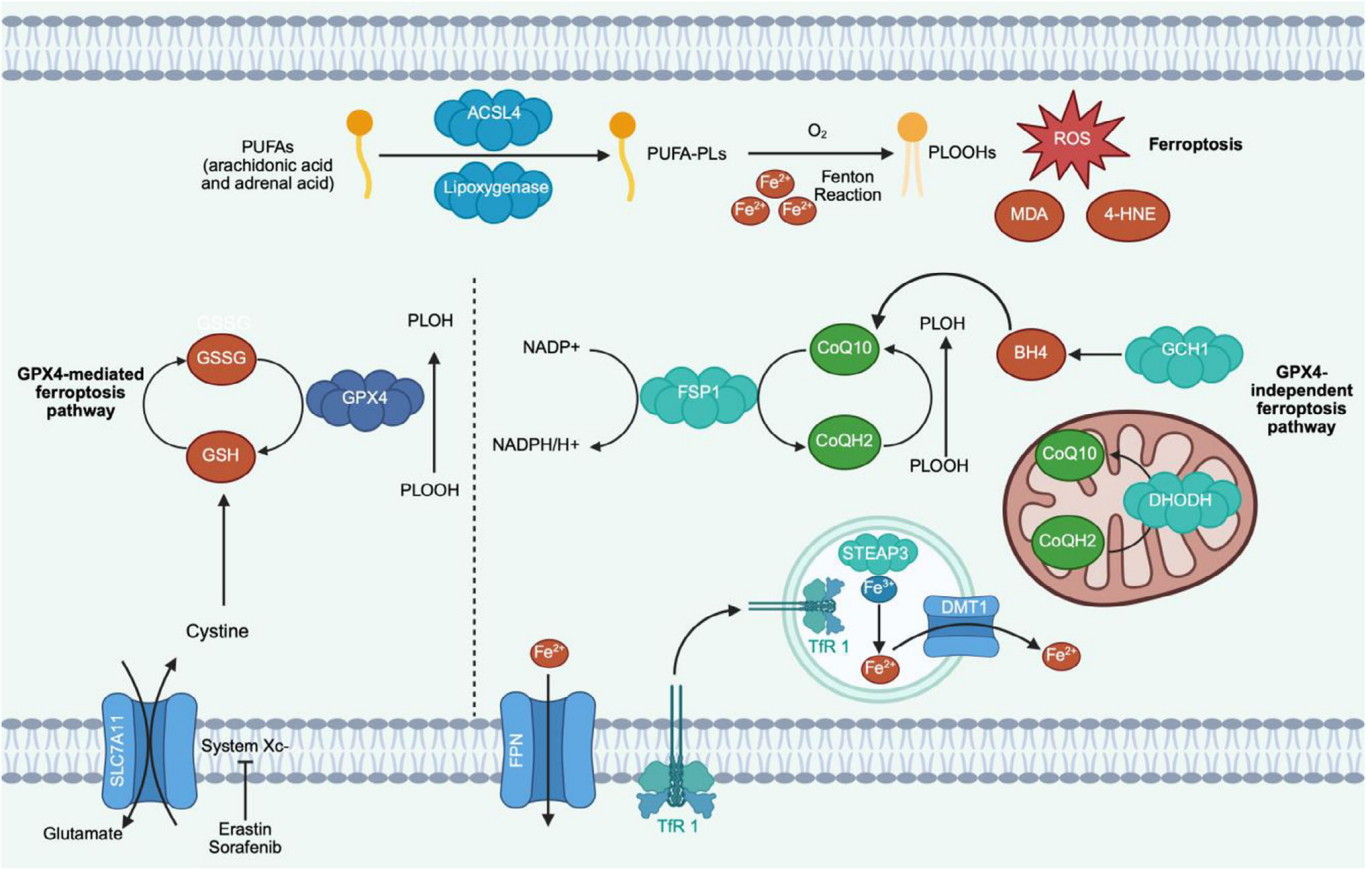
A brief overview of ferroptosis and two pathways to inhibit ferroptosis. The schematic shows that ferroptosis is performed by phospholipid peroxidation, a process that depends on ROS, PUFAs, PUFA‐PLs, and Fe^2+^, among others. GPX4‐mediated ferroptosis pathway is mainly mediated by GPX4‐mediated GSH/GSSG interaction and PL‐OOH reduction to produce the corresponding P‐LOH. The GPX4‐independent ferroptosis pathway is mainly involved in the FSP1/CoQ10/NADPH axis and the GCH1/BH4 axis, in addition to mitochondrial DHODH/CoQ10. Finally, it is also described that Fe^2+^ can be transported throungh FPN transporters, as well as the TfR1/STEAP3/DMT1 pathways.

With the development of research, the mechanism of iron‐dependent lipid peroxidation in cells, including mitochondria, has been gradually revealed. Intracellular ferroptosis‐related non‐GPX4 pathways, including FSP1‐CoQ10, GCH1‐BH4, and DHODH‐CoQ10 pathways, and the typical enzymatic System with System Xc‐GSH‐GPX4 as the core, together regulate the balance between oxidative damage and antioxidant defense during ferroptosis. Ferroptosis is the intersection of multiple metabolic pathways in cells. Amino acid metabolism, lipid metabolism, iron metabolism, and other metabolic pathways regulate the sensitivity of cells to ferroptosis (Figure 1).

### 2.3 Crosstalk between Ferroptosis and Other Cell Death Pathways

In the process of ferroptosis, several organelles absorb and release iron ions and lipids. Therefore, there could be extensive crosstalk with autophagy. Among them, the excessive activation of selective autophagy, including ferritinophagy, clockophagy, lipophagy, and chaperone‐mediated autophagy, facilitates ferroptosis by degrading ferritin, circadian protein, lipid droplets, and GPX4, respectively [39]. Ferritinophagy is a process involving the promotion of selective autophagy of ferritin by nuclear receptor co‐activator 4 (NCOA4), which requires the recognition of ferritin by NCOA4 and then its delivery to autophagosomes [40]. Clockophagy is the selective autophagic degradation of the circadian clock regulator ARNTL/BMAL1. Clockophagy promotes lipid peroxidation and subsequent ferroptosis by blocking HIF1A‐dependent fatty acid absorption and lipid storage [40]. Lipophagy, which is defined as the autophagic degradation of intracellular lipid droplets, is another type of autophagy that crosstalks with ferroptosis. RAB7, as a key molecule, mediates lipid droplet digestion and releases free fatty acids. Then it promotes mitochondrial oxidation and ferritin proliferation [41]. Chaperon‐mediated autophagy (CMA) is a cellular lysosomal degradation mechanism regulated by HSP90. CMA effectively promotes the occurrence of ferroptosis by facilitating the degradation of GPX4 [42].

In addition to autophagy, apoptosis is also interfered with by ferroptosis. Studies have shown that lipid peroxidation can also induce the occurrence of apoptosis [43]. Through the NF‐κB pathway and the antiapoptotic protein BCL‐2, lipid peroxides effectively interfere with the apoptotic behavior of cells. Furthermore, lipid peroxidation products form complexes with P38, JNK, and ERK to activate mitogen‐activated protein kinase (MAPK) and Caspase signaling to initiate cell apoptosis [44].

### 2.4 Diagnostic Markers of Ferroptosis

Through the characteristics of ferroptosis, the occurrence of ferroptosis under physiological and pathological conditions can be determined. Furthermore, the role of ferroptosis in female health and disease was further explored. At present, there are four markers considered for detecting ferroptosis behavior, including lipid peroxidation, mitochondrial morphological changes, gene expression changes, and TFR1 relocalization [45].

First, the lipid peroxidation behavior in ferroptosis can be detected by five methods, including MDA, 4‐HNE staining, thiobarbituric acid reactive substances (TBARS), BODIPY 581/591 C11 fluorescent probes, and lipidomics methods. Second, during the process of ferroptosis, multiple organelles are involved. Among them, the morphological changes of mitochondria, characterized by contraction, increased density, and reduced crystals, are regarded as the characteristic morphology of ferroptosis and can be observed using a transelectric microscope [45, 46]. Third, specific gene expression changes can be detected in ferroptotic cells, such as increased *CHAC1, PTGS2, SLC7A11*, and *ACSL4* [29, 45]. Finally, the repositioning of TFR1, which introduces extracellular iron into cells through endothelial cell proliferation, helps to reduce the burden required for iron cleavage. The negative iron pool has been proven to be a marker of iron cleavage. The transfer of TFR1 from the region surrounding Golgi to the plasma membrane can be observed by staining with a 373‐FMA antibody [47].

To exactly determine the occurrence of ferroptosis, several markers are required, and these markers must be detected before cell death. Choosing an appropriate time point is very important for detecting the markers of ferroptosis. Among these markers, lipid peroxidation is indispensable, while other indicators may not be fully detectable.

## 3 Ferroptosis in Obstetric Diseases

The placenta is an important organ for maternal‐fetal material exchange and immune tolerance [48, 49]. Abnormal placental structure and function affect the normal pregnancy process. Trophoblast cells are rich in iron, and the risk of ischemia/reperfusion and membrane lipid peroxidation exposure is increased during placental development, which makes trophoblast cells prone to ferroptosis [50]. Kajiwara et al. showed that the characteristic membrane bleb occurs in trophoblast cells during ferroptosis, while the nuclei are normal in morphology, without chromatin condensation and mitochondrial atrophy [51]. Studies have shown that trophoblast cells, such as human primary trophoblast (PHT) and BeWo cell lines, have higher ferroptosis sensitivity than other commonly used cell lines [51, 52]. As an important marker of placental dysfunction, oxidative stress and lipid peroxidation toxicity increase the induction of ferroptosis‐related pathways, which play a key role in trophoblast dysfunction that are closely related to diseases such as gestational diabetes mellitus (GDM), preeclampsia (PE), preterm birth, and miscarriage.

### 3.1 Ferroptosis and GDM

GDM is a type of normal glucose metabolism before pregnancy, and abnormal glucose tolerance appears or is detected for the first time during pregnancy [53, 54, 55, 56], which is one of the common complications of pregnancy. The main reason for the pathological changes of the placenta in these patients is the imbalance of oxidative stress. When the placenta undergoes oxidative stress imbalance, the lipid metabolism of placental tissue is destroyed, leading to further occurrence of ferroptosis [15].

During pregnancy, there is a significant increase in iron requirements due to the expansion of maternal blood volume and fetal growth and development [57, 58]. To prevent the occurrence of anemia during pregnancy, the World Health Organization (WHO) recommends the routine administration of iron supplements to pregnant women [59]. However, an increasing number of studies have revealed a potential association between excessive iron stores during pregnancy and the development of GDM.

Cytological studies have also confirmed that in a high‐glucose environment, GPX4 expression in trophoblast cells is downregulated, lipid peroxidation is activated, ROS and MDA concentrations are increased, and ferroptosis occurs in trophoblast cells [60]. Furthermore, an increasing number of clinical studies have identified potential associations between iron‐related indicators and GDM. An early study found that iron‐related indicators showed significant changes in the GDM patient group compared with the control group [61]. Moreover, one study shows that compared with healthy pregnant women, the blood GSH content of patients with GDM is significantly decreased, and the MDA level is increased, while the changing trend of both is significantly positively correlated with the disease degree of GDM [60]. GSH is an effective marker of oxidative stress and an important indicator used to understand the extent of ferroptosis. A recent prospective study also demonstrated a correlation with significance between iron overload and GDM. Researchers compare the ferritin levels and ferri regulation concentration in GDM patients and healthy pregnant women and find that ferritin levels in the first and second trimesters are positively associated with the risk of developing GDM, and pregnant women with high ferritin levels in the first trimester have a 21% higher risk of developing GDM than those with normal ferritin levels [62].

Given the close association between iron‐related indicators and GDM, iron‐related indicators may be used as effective factors for predicting or treating GDM in clinical practice. A prospective cohort study found that increased plasma ferritin concentrations in the first half of pregnancy and iron supplementation of 60 mg/day or more during pregnancy were independently associated with an increased risk of GDM [63]. Liu et al.systematically summarized the association between hemoglobin and ferritin with GDM [57]. However, further trials are needed to validate the feasibility of iron‐related indicators as predictive indicators for GDM.

Additionally, the combined targeting of iron‐related damage may be an effective strategy for treating GDM. For example, a study by Han et al. reports that [64], in response to high concentrations of glucose and ferroptosis inducers, the expression of sirtuin 3 (SIRT3) in trophoblast cells is increased, consequently activating ferroptosis. *SIRT3* deficiency inhibits the elevation of GPX4 and thus ferroptosis, which may be a new drug target. Galana et al. find that melatonin may reduce the pathological damage of GDM by inhibiting ferroptosis‐related signaling molecules, thus benefiting the treatment of GDM [65]. Adiponectin is an insulin sensitizer, which is highly expressed in adipose tissue and can enhance the inhibitory effect of insulin on glyconeogenesis. In the study by Zheng et al. [66], it is found that adiponectin can inhibit blood sugar and lipid levels in mice with GDM, correcting ferroptosis induced by fatty acid oxidation and peroxide imbalances, restore carnitine palmitoyl transferase 1 (CPT‐1, long‐chain fatty acids enter the mitochondria for beta‐oxidation rate‐limiting enzyme) activity, thereby ameliorating placental damage of GDM. Zhang et al. further reported that elevated glucose transporter 1 (GLUT1) expression inhibits AMPK phosphorylation and decreases acetyl‐CoA carboxylase phosphorylation, which in turn enhances lipid synthesis and ferroptosis, and ultimately promotes fetal growth restriction associated with gestational diabetes mellitus [16].

**FIGURE 2:**
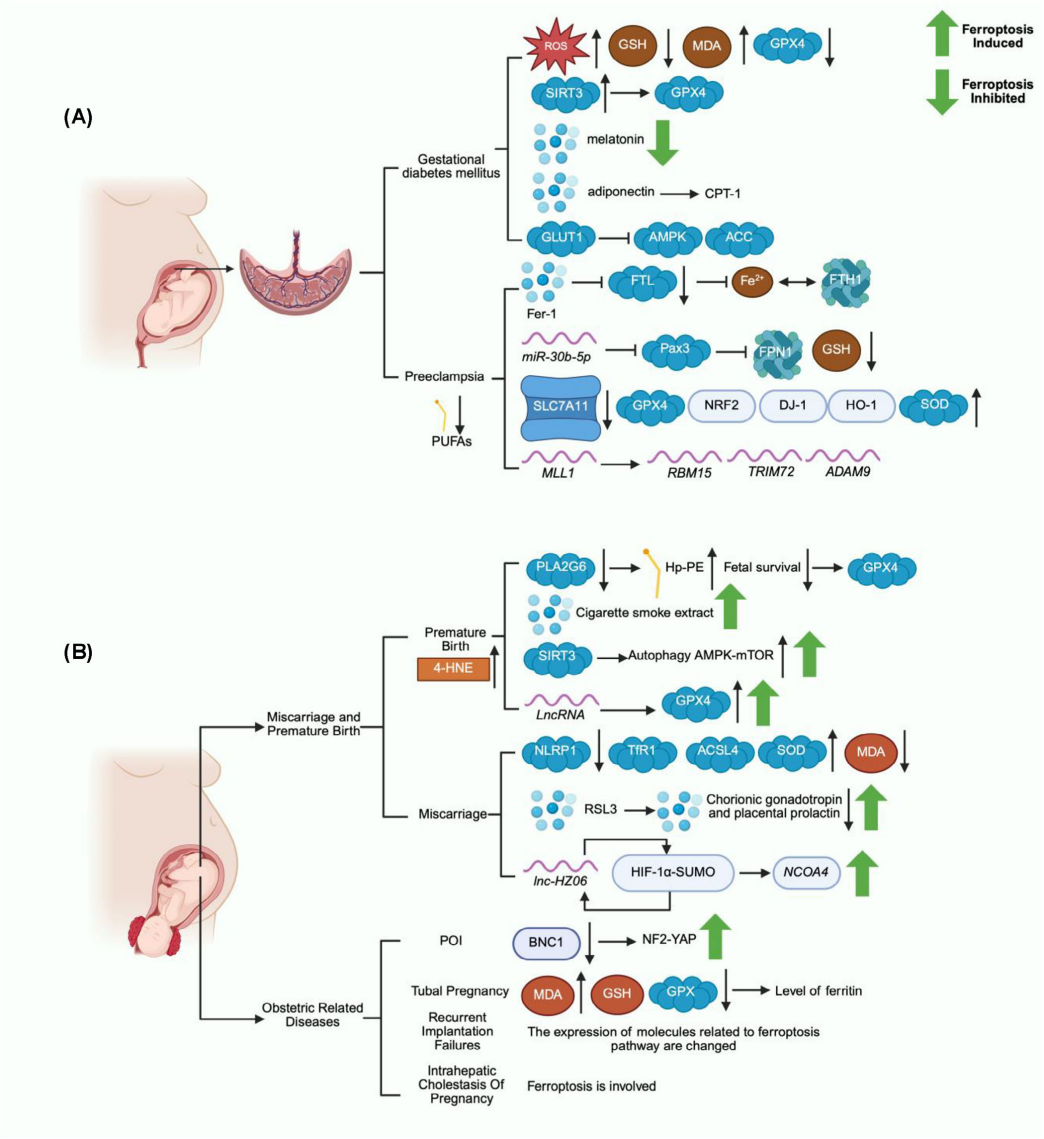
Gestational dysfunction occurs during a woman's pregnancy. (A) Association between preeclampsia, gestational diabetes mellitus, and ferroptosis. (B) Association between miscarriage, preterm birth, and ferroptosis. The relationship between primary ovarian insufficiency, tubal pregnancy, repeated implantation failure, intrahepatic cholestasis of pregnancy, and ferroptosis was also described. In addition, the induction and inhibition of the ferroptosis are indicated by the up and down direction of the green arrows.

In summary, the occurrence and development of GDM are accompanied by the behavior of ferroptosis (Figure 2A). Therefore, regulation of ferroptosis has great research value for the protection of GDM patients and provides a new direction for clinicians.

### 3.2 Ferroptosis and PE

PE is a condition characterized by hypertension before 20 weeks of gestation and may have one other related complication (proteinuria, uteroplacental dysfunction, maternal organ dysfunction), and the main causes are insufficient trophoblast migration and invasion ability and uterine spiral artery remodeling disorder [67, 68, 69, 70, 71]. Placental regional dysplasia and hypoxic‐ischemic environment lead to ROS accumulation, and PE shows increased oxidative stress consistent with ferroptosis, and dysregulated iron and lipid metabolism [72]. Ferroptosis regulates trophoblast function at different levels by regulating iron metabolism, oxidative stress, GPX4 function, and inflammatory response, and participates in the pathophysiology of PE [73, 74].

The level of placental PUFAs in PE patients is significantly higher than that in pregnant women with normal blood pressure, and the level of lipid metabolism is related to the severity of PE [75, 76]. The accumulation of lipid peroxidation metabolites in placental tissues and serum is accompanied by increased oxidative stress, imbalance of the cellular antioxidant system, and dysregulation of iron homeostasis [1, 77, 78, 79]. The expression profiles of key genes in ferroptosis show dynamic changes during the occurrence and development of PE‐placental function impairment and disease. Increased labile iron content has been detected in placental tissues of PE patients, while the expression of ferritin heavy chain 1 (FTH1), ferritin light chains (FTL), and ferroportin 1 (FPN1) is decreased [80, 81, 82]. In the report by Yang et al. [82], the expression of FTL is downregulated in the placental villi and decidua tissues of PE patients, and FTL is specifically expressed in the cytotrophoblast of the human placenta in the first and third trimesters. As the main transport form of intracellular iron, Fe^2+^ binds to FTH1 and is maintained by FTL. Some of the pathologic phenotypes of PE, such as impaired spiral artery remodeling, trophoblast cell invasion, and migration dysfunction, were seen in pregnant rat models with *FTL* knockdown, accompanied by changes in ferroptosis metabolism. However, after administration of Ferrostatin‐1 (Fer‐1), this phenotype can be significantly blocked, and the invasion and migration capacity of trophoblast cells can be improved [82]. Similarly, in vitro experiments have shown that Fer‐1 can also ameliorate hypoxia‐induced motility reduction of HTR‐8/SVneo and TEV‐1 cells [81]. These results suggest that ferroptosis plays an important role in the development of PE, and the introduction of specific Fer‐1 can reverse this iron‐dependent disorder of lipid metabolism. Zhang et al. reported that miR‐30b‐5p can trigger ferroptosis in trophoblast cells under hypoxic conditions by downregulating the transcription factor paired box protein 3 (Pax3) and its downstream target FPN1 in hypoxia‐induced cell and animal models. Moreover, the knockdown of miRNA can reverse the ferroptosis phenotype of PE rat placenta by regulating the iron export process and improving blood pressure, urinary protein concentration, and embryo survival rate [81]. The activity of GSH and the expression of SLC7A11in the placenta of PE patients are decreased, and the Fe^2+^ level and blood pressure indicators to evaluate the severity of PE are changed. Hypoxia can stimulate the accumulation of ROS. With the depletion of GSH and the accumulation of Fe^2+^, the oxidative capacity of ROS is further enhanced, leading to the production of large amounts of lipid peroxides and increased ferroptosis sensitivity. Oxidative stress is the main cause of the adverse consequences of PE. The expression of oxidative stress‐related indicators in placental tissue, such as GPX4, nuclear factor erythroid 2‐related factor 2 (NRF2), and human Parkinson gene 7 (DJ‐1) is upregulated, and DJ‐1 protein expression is positively correlated with NRF2 and GPX4 protein expression in placental tissue [77]. It was found that hypoxia can not only promote the translocation of NRF2 to the trophoblast nucleus but also cause the activation of NRF2/heme oxygenase‐1 (HO‐1) signal transduction, which exacerbates the oxidative stress of trophoblast cells with PE [83]. Cell redox signal sensor DJ‐1 plays a vital role in activating and stabilizing NRF2. After translocation to the nucleus, NRF2 can bind to antioxidant elements and induce the expression of downstream antioxidant enzymes, including HO‐1, GPX4, and superoxide dismutase [84]. The upregulated expression of DJ‐1 in BeWo cells may inhibit ferroptosis of trophoblast cells through the NRF2/GPX4 signaling pathway, and play a protective role in the pathophysiology of trophoblast dysfunction in patients with PE [83]. With transmission electron microscopy, trophoblast cells in PE patients show typical features of ferroptosis, while intracellular organelles, including mitochondria, are generally swollen[84], suggesting that this characteristic manifestation of nonferroptosis may be the result of multiple pathways involved in trophoblast death. It has been found that a hypoxic condition will lead to ferroptosis activation and cause sEV‐mediated extrusion of harmful lipid peroxides from trophoblast cells into the circulation, thereby affecting maternal dysfunction in PE [85]. In the study by Li et al. [86], it was found that MLL1 can promote ferroptosis in placental trophoblast and aggravate PE symptoms through epigenetic regulation of the RBM15/TRIM72/ADAM9 axis. Moreover, the PPARγ/Nrf2 signaling has been found to affect ferroptosis by regulating lipid oxidation but not SREBP1‐mediated lipid synthesis, thereby alleviating PE development [87].

In conclusion, ferroptosis behavior was observed in placental tissues and cell and animal models of PE (Figure 2B), suggesting ferroptosis regulation may contribute to intervention in PE disease.

### 3.3 Ferroptosis and Preterm Birth and Miscarriage

Preterm birth, defined as delivery within 37 weeks or 259 days of gestation, is the leading cause of high maternal complications and mortality in both developed and developing countries [88, 89, 90, 91, 92]. Miscarriage is usually defined as the termination of a pregnancy before the embryo or fetus is viable. It is estimated that there are 23 million miscarriages worldwide each year, 44 per minute [93, 94, 95]. At present, the incidence of miscarriage and preterm birth is still rising, and the events leading to miscarriage and preterm birth are still not fully understood. Other trophoblastic disease is also found in the pathophysiological process of lipid evidence of toxicity, and nourish cells’ GSH antioxidant capacity and GPX4 lipid peroxidation damage repair ability [64, 96, 97].

Placental lipid metabolome dysregulation, including increased hydroxy‐peroxidized phosphatidylethanolamine (Hp‐PE) and 4‐HNE, characteristic of ferroptosis, has been reported in spontaneous preterm birth [52]. Phospholipase A2 group *VI* (PLA2G6) is universally expressed in the human placenta, which can metabolize Hp‐PE to oxidized fatty acids and alleviate ferroptosis caused by GPX4 inhibition in vitro or hypoxia/reoxygenation injury in vivo. According to recent reports, the *PLA2G6* gene or specific inhibitors can increase the percentage of lactate dehydrogenase release and the fluorescence intensity of lipid peroxides in BeWo cells. Hp‐PE accumulation and decreased fetal survival are detected in the placenta of *Pla2g6* knockout mouse models [52]. In particular, GPX4 expression is decreased and PLA2G6 expression is increased during PHT cell differentiation, further suggesting that PLA2G6 may play a protective compensatory role in trophoblast ferroptosis caused by GPX4 inactivation. In the opinion of Guan et al. [98], it is believed that cigarette smoke extract can induce ferroptosis and peroxisome dysfunction in rat placental trophoblast cells, leading to adverse pregnancy outcomes [99]. Han et al. believe that SIRT3, a major regulatory molecule of mitochondrial function and lipid metabolism homeostasis, can activate autophagy and promote the MAPK‐mammalian target of rapamycin (MAPK‐mTOR) pathway, then ferroptosis was induced in trophoblast cells, suggesting that ferroptosis in trophoblast cells may interact with autophagy and be related to mitochondrial dysfunction in trophoblast cells [64]. Furthermore, posttranscriptional levels such as *LncRNAs* may play a regulatory role in GPX4‐mediated ferroptosis in trophoblast cells [100].

In the oxidative stress miscarriage model, ferroptosis of trophoblast and inflammation body Nod‐like receptor protein 1 (NLRP1) is activated, and silencing of NLRP1 leads to the increase of Transferrin receptor protein 1(TfR1), ACSL4, SOD expression, and the decrease of MDA content [96]. On the other hand, ferroptosis inhibitors can inversely downregulate the expression of inflammatory bodies and downstream inflammatory factors and participate in the pathophysiological process of placental dysfunction. In addition, ferroptosis inducer RSL3 may induce a decrease in the release of human chorionic gonadotropin and placental prolactin, increasing ferroptosis damage in the trophoblast by affecting trophoblast differentiation [52]. In addition, recent studies have shown that *Lnc‐HZ06* and HIF1‐SUMO form a positive feedback loop [101]. This cycle is upregulated particularly in hypoxic trophoblast cells, villi tissue from patients with recurrent miscarriage of unknown etiology, and placenta tissue from hypoxia‐treated mice. Ferroptosis and miscarriage can be further induced by upregulation of HIF1α‐SUMO‐mediated transcription of *NCOA4*.

The dysfunction of the above trophoblast cells can lead to premature delivery and miscarriage (Figure 2B), and all these examples show that there is a strong relationship between ferroptosis and miscarriage. In the future, maternal protection might be achieved by regulating the expression of ferroptosis‐related proteins.

### 3.4 Ferroptosis and Other Obstetric‐Related Diseases

Recently, it has been found that ferroptosis is also closely related to other obstetric diseases. Primary ovarian insufficiency (POI) is a form of female infertility, defined as premature ovarian failure before the age of 40, characterized by a clinical syndrome of ovarian dysfunction with amenorrhea [102, 103, 104]. Premature ovarian failure can lead to infertility, serious disturbances in daily living, and long‐term health risks in age‐appropriate women [105, 106]. However, the underlying mechanism remains a mystery. In one study, it was found that Basonuclin 1 (BNC1) plays a key role in ovarian reserve [107]. During follicular development*, BNC1* can maintain oocyte redox homeostasis and lipid metabolism, while a deficiency of *BNC1* can lead to excessive follicular atresia and premature activation. Mechanistically, when *BNC1* is deficient, it triggers oocyte ferroptosis through the NF2‐YAP pathway, ultimately leading to POI. Thus, POI induced by *BNC1* mutations can be significantly reduced after inhibition of YAP signaling or ferroptosis with drugs. In the villus tissues of patients with tubal pregnancy, MDA content is increased, the level of GSH and GPX is reduced, and the expression level of ferritin is increased [108], suggesting ferroptosis occurs in tubal pregnancy. Some studies have sampled endometrial tissue from patients with recurrent implantation failures and those with normal fertility, then isolated primary cells for protein extraction. It is found that the expression of molecules related to the ferroptosis pathway is changed in patients with repeated implantation failure [109]. Through bioinformatics analysis, more studies have found that ferroptosis is also involved in the occurrence and development of intrahepatic cholestasis in pregnancy [110].

All of the above reports suggest that ferroptosis is associated with the onset and development of various diseases associated with pregnancy in women (Figure 2A). In the future, ferroptosis may become an effective direction for early screening and treatment of difficult and miscellaneous diseases.

## 4 Ferroptosis in Gynecological Diseases

Since the discovery of ferroptosis, there has been much interest in the mechanism of action between ferroptosis and tumor biological behavior. Some studies have reported that there is a clear relationship between ferroptosis and tumor‐related signaling pathways [111, 112, 113]. Among them, research on gynecologic tumors and ferroptosis mainly includes ovarian cancer, cervical cancer, endometrial cancer, and breast cancer (BC) [114, 115, 116, 117]. In addition, ferroptosis can also be found in some common cases of endometriosis or polycystic ovary syndrome [117, 118].

### 4.1 Ferroptosis and Ovarian Cancer

Ovarian cancer is one of the deadliest malignancies in women, and its five‐year relative survival rate is less than 50 percent due to inadequate early detection methods and high recurrence rates [119, 120, 121, 122, 123]. At present, the simple standard treatment used in most cases does not produce good results. Therefore, there is still a need to provide more effective treatments [124].

Clinically, the first‐line chemotherapy for ovarian cancer is platinum drugs combined with paclitaxel, but chemotherapy resistance has become a difficult problem. Deacetylase 5 inhibits cisplatin‐induced DNA damage by regulating Nrf2/heme HO‐1 signaling pathway in a ROS‐dependent manner, resulting in cisplatin resistance in ovarian cancer cells (SKOV‐3 and CAOV‐3) [125]. Previous studies have found that ferroptosis is involved in the regulation of platinum‐resistant ovarian cancer cells, and the survival of the platinum‐resistant cell population marked by frizzled transmembrane receptor 7 (FZD7) depends on the FZD7/ β‐catenin /Tp63/GPX4 signaling pathway [126]. The ovarian cancer cell line OVCAR5, after FZD7 knockdown, is highly sensitive to ferroptosis. In addition, the expression of GPX4 and SLC7A11 in platinum‐resistant ovarian cancer cell lines SKOV3 and A2780 can be downregulated by supermagnetic nano‐iron oxide (SPIO) human serum, which can increase the level of ROS in cells and induce ferroptosis in cancer cells [127]. For paclitaxel‐resistant cell lines, Erastin may inhibit the activity of multidrug resistance gene 1/P glycoprotein to promote paclitaxel drug efflux, thereby reversing docetaxel resistance in paclitaxel‐resistant A2780 cells [127]. In ovarian clear cell cancer cells that are prone to paclitaxel and platinum resistance, iron–sulfur cluster synthesis can be restricted by inhibiting cysteine uptake, thus disrupting the electron transport chain. The destruction of the electron transport chain will lead to iron overload and mitochondrial damage and ultimately induce cell ferroptosis [128]. These results suggest that more inhibitors of related signaling pathways and ferroptosis inducers targeting specific resistance genes can be developed in the future, and the specific mechanisms between developed drugs and resistant strains can be explored. Perhaps the induction of ferroptosis could break the current therapeutic dilemma faced by patients who are resistant to platinum or paclitaxel.

Ferroptosis inducers can promote ferroptosis in ovarian cancer cells when used alone or in combination with other drugs [129]. One study found the presence of a single chain type I ribosome‐inactivating protein (MAP30) in bitter melon that works synergistically with cisplatin [130]. This synergistic effect can cause Ca^2+^ influx in ovarian cancer cell lines including OVCA43, ES2, HEY, and HEYA8, causing cytosolic oxidative stress and mitochondrial dysfunction, and finally inducing the occurrence of ferroptosis in ovarian cancer cells. Another study also shows that Erastin combined with cisplatin can increase the expression of ACSL4 and cyclooxygenase‐2 (COX‐2) and decrease the expression of GPX4 and FTH1 in HEY cells and ovarian cancer tissues, thus promoting ferroptosis in HEY cells and inhibiting tumor growth [131]. The simultaneous intraperitoneal injection of MAP30 and cisplatin in ES2‐bearing nude mice can reduce tumor weight, tumor number, and average abdominal water volume significantly. In the study by Hong et al. [132], the HEY cells are inoculated into nude mice. Sulfasalazine, Olaparib, or a combination of the two drugs is administered. The results show that the tumor is resistant to Olaparib. Although Sulfasalazine alone does not significantly increase efficacy, the combination of the two drugs increases tumor sensitivity to Olaparib. Moreover, the combined drugs can effectively inhibit the occurrence and development of tumors, prolong the survival time of nude mice, but do not significantly reduce the body weight of nude mice, indicating that nude mice can tolerate the toxicity of the combined drugs. These results all suggest that the combination of ferroptosis inducers and conventional drugs can have a better antitumor effect, and there is no cumulative toxic side effect. Sodium citrate is found to inhibit glycolysis and induce apoptosis through the CAMKK2/AKT/mTOR/HIF1α signaling pathway mediated by Ca^2+^ chelation [133]. In addition, Ca^2+^ chelation by sodium citrate promotes NCOA4‐mediated ferroptosis and attenuates mitochondrial Ca^2+^ uptake by inhibiting the activation of mitochondrial Ca^2+^ uniporter (MCU) activity, resulting in high ROS levels within mitochondria. Ultimately, these changes induce the occurrence of ferroptosis, providing new ideas for the treatment of ovarian cancer and overcoming drug resistance.

It has also been found that ferroptosis‐related genes are closely related to the prognosis of ovarian cancer patients. It has also been found that there are six ferroptosis‐related hub genes (driver genes *DNAJB6, BACH1*, and *ALOX12*, suppressor genes *RB1*, and marker genes *SELENOS* and *STEAP3*) in ovarian cancer, which have been confirmed to be closely related to the disease progression and prognosis of ovarian cancer patients [134]. Chen et al. have the opinion that cytochrome b reductase 1 (CYBRD1) is highly expressed in ovarian cancer and mediates the occurrence and development of ovarian cancer by affecting iron uptake and ferroptosis‐related signaling pathways [135]. In addition, it is confirmed in case studies that CYBRD1 expression is remarkably associated with lymph node metastasis, advanced stage, low differentiation, and poor prognosis. Additionally, in the study by Chai et al. [136], *hsa_circ_0001546* is found to be downregulated in epithelial ovarian cancer (EOC). *Hsa_circ_0001546* directly binds *14‐3‐3* to form the *hsa_circ_0001546/14‐3‐3* complex, further recruits the kinase CAMK2D, induces abnormal phosphorylation of Tau protein at Ser324, resulting in Tau aggregation‐dependent lipid peroxides (LPO) accumulation and ferroptosis, and ultimately prevents the progression of EOC. It also confirmed a strong relationship between ferroptosis and ovarian cancer.

**FIGURE 3:**
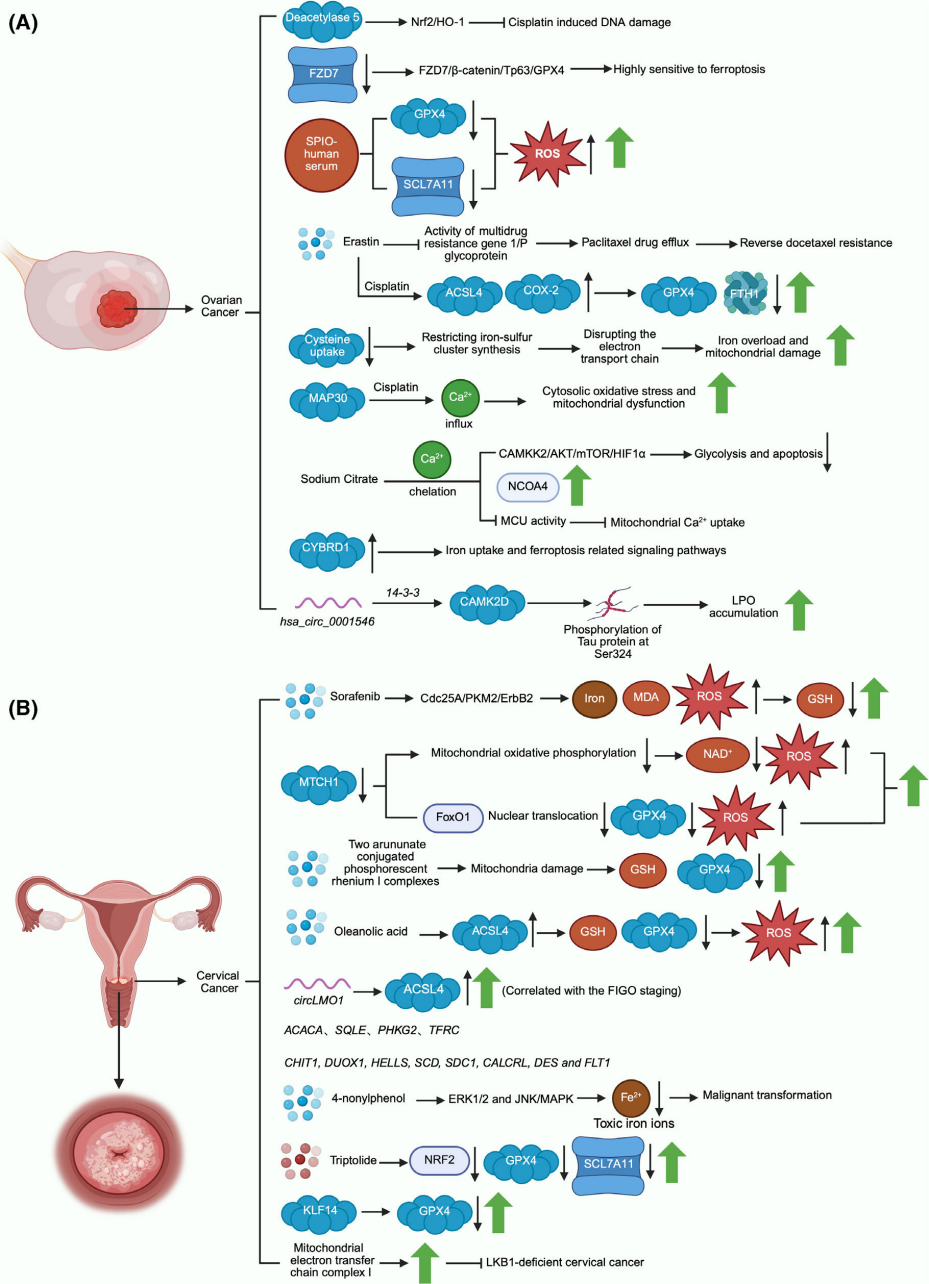
A graph of female reproductive system diseases. The abnormal ovarian tissue in (A) is ovarian cancer tissue, focusing on the relationship between ovarian cancer and ferroptosis. The abnormal tissue of the cervix in (B), cervical cancer tissue, mainly depicts the relationship between cervical cancer and ferroptosis.

According to the above studies, the current research on ferroptosis and ovarian cancer is relatively comprehensive (Figure 3A). Ferroptosis is closely related to the development and prognosis of the disease and is involved in the regulation of drug resistance in drug‐resistant ovarian cancer cell lines. In addition, ferroptosis inducers combined with conventional chemotherapy drugs can more effectively inhibit the growth of cancer cells and cancer tissues without doubling the toxic effects. All these can provide a new direction for the high recurrence rate and drug resistance rate of ovarian cancer in clinical treatment. Moreover, the combination of modern medicine and materials science, such as SPIO‐human serum, shows that ferroptosis inducers also have strong therapeutic potential and may play an unexpected role in the treatment of ovarian cancer.

### 4.2 Ferroptosis and Cervical Cancer

As one of the gynecological malignant tumors, cervical cancer has the highest incidence among women of childbearing age [84, 137, 138]. Despite the existence of preventive vaccines, morbidity, and mortality remain a problem in developing countries. According to the International Federation of Obstetrics and Gynecology (FIGO), patients with disease in the IB‐IIA stage have a recurrence risk of 10–20%; For patients at the IIB‐IVA stage, the risk of recurrence is 50–70%. Patients with distant metastasis and local uncontrolled disease recurrence have a worse prognosis [139, 140].

Ferroptosis inducers can promote ferroptosis in cervical cancer cells. According to Wang et al.’s research [141], Sorafenib can increase iron content, MDA and ROS levels but decrease GSH levels in cervical cancer cell lines SiHa and CaSki cells through the cell division cycle enzyme 25A (Cdc25A)/M2 pyruvate kinase (PKM2)/tyrosine kinase receptor 2 (ErbB2) signaling pathway, so that the cancer cells show the typical characteristics of ferroptosis. It has been found that mitochondrial carrier 1 (MTCH1) is the central mediator of mitochondrial‐mediated ferroptosis in cervical cancer [142]. When MTCH1 is deficient, mitochondrial oxidative phosphorylation is disrupted, and ROS in mitochondria is increased by decreasing nicotinamide adenine dinucleotide (NAD^+^) levels. This mitochondrial autonomous event triggers a retrograde signal from mitochondria to the nucleus involving reduced FoxO1 nuclear translocation and subsequent downregulation of transcription and activity of the key antiferroptosis enzyme GPX4, thereby elevating ROS and ultimately triggering ferroptosis. Most notably, targeted MTCH1, when combined with Sorafenib, effectively and synergistically inhibited cervical cancer growth in a nude mouse xenograft model by actively inducing ferroptosis. In Ye et al.’s study [143], two artesunate‐conjugated phosphorescent rhenium I complexes are designed, which could damage the mitochondria of HeLa cells, causing GSH, GPX4 inactivation, and lipid peroxide accumulation, and inducing ferroptosis of HeLa cells. By upregulating the expression of ACSL4 and downregulating the expression of GPX4 and GSH in HeLa cells, oleanolic acid in herbaceous plants causes the accumulation of lipid peroxidation and ROS, resulting in ferroptosis of HeLa cells that inhibits the growth of cancerous tissue in HeLa‐cell bearing nude mice [144]. At present, while these studies mainly focus on cell experiments with a single selected cell line, more researches are needed to verify.

Ferroptosis‐related genes can also be used as indicators to evaluate the prognosis of cervical cancer patients. Ou et al.’s study holds the view that *circular RNA LMO1 (circLMO1*) can upregulate the expression of ACSL4, promote ferroptosis of CaSki cells, and reduce the expression of circLMO1 in cervical cancer tissues, promoting the invasion and proliferation of cancer cells [145]. They also suggest that *circLMO1* expression levels are correlated with the FIGO staging of cervical cancer and may be a candidate therapeutic target or a potential prognostic marker for cervical cancer. In some studies [145], seven kinds of long noncoding RNAs related to ferroptosis are screened from the Cancer Genome Atlas (TCGA) and ferroptosis database, and the samples are divided into a low‐risk group and a high‐risk group according to the median risk score. The results show that the mortality rate of cervical cancer patients in the low‐risk group is lower than that in the high‐risk group. The results of time‐dependent receiver‐operating characteristic curve evaluation suggest that these seven long noncoding RNAs are major risk factors for patients with cervical cancer and can be used as potential markers to predict the prognosis of patients with cervical cancer [35, 146]. Another study analyzes the ferroptosis‐related genes of 60 patients with cervical cancer and their relationship with prognosis through the TCGA database [147], and finally screens out four differential genes (*ACACA, SQLE, PHKG2, TFRC* genes). In the Xing et al. study [148], eight differentially expressed genes related to ferroptosis and immunity (*CHIT1, DUOX1, HELLS, SCD*, *SDC1*, *CALCRL*, *DES*, and *FLT1* genes) are screened out, and the cervical cancer prediction models constructed are all independently correlated with overall survival. It is noteworthy that some of these genes are also differentially expressed in endometrial cancer or ovarian cancer studies, such as the *TFRC* gene is highly expressed in the proteomics of patients with early‐stage low‐grade endometrial cancer [149], and the *SCD* gene is highly expressed in ovarian cancer tissues and cells [150]. Whether these common differential genes can be used as universal tumor markers deserves further exploration and verification. In the report of Zhang et al. [151], it was found that long‐term 4‐nonyl phenol exposure enhances the antioxidant capacity of cells by enhancing the phosphorylation of ERK1/2 and JNK/MAPK pathways, and promotes the removal of toxic iron ions, which ultimately mediates the malignant transformation of cervical epithelial cells. It has also been reported that triptolide can significantly reduce the expression of NRF2, leading to corresponding inhibition of NRF2 downstream targets GPX4 and SLC7A11, and finally promoting ferroptosis in cervical cancer cells [152]. In the study by Ye et al. [153], it was found that KLF14 binds to the promoter of GPX4, inhibits its transcriptional activity, and thus reduces its expression, which helps to promote ferroptosis of cervical cancer and plays an antitumor role. In the research of Mao et al. [154], it is confirmed that mitochondrial electron transfer chain complex I has many functions in regulating ferroptosis, and the possibility of treating LKB1‐deficient cancers (such as cervical cancer and ovarian cancer, etc.) by ferroptosis induction is put forward.

At present, the research on ferroptosis and cervical cancer mainly focuses on the mining and analysis of bioinformatics databases. The correlation between these ferroptosis‐related genes and cervical cancer patients is mainly reflected in the incidence and prognosis (Figure 3B). If it can be verified by a large number of clinical cases throughout treatment, then in the future, these genes are highly likely to become markers for monitoring clinical treatment effects and predicting prognosis. However, studies on the mechanism of ferroptosis in cervical cancer are relatively scarce, and the conclusions drawn have limited reference value for clinical practice. Therefore, a large number of both in vivo and in vitro experiments are needed to deeply study the regulatory mechanism of ferroptosis in cervical cancer in the future, and ferroptosis may become a new direction for the treatment of cervical cancer.

### 4.3 Ferroptosis and Endometrial Cancer

Endometrial cancer is a gynecological tumor that occurs in the lining of the uterus, and its incidence is currently steadily increasing [155, 156, 157, 158, 159]. At present, most cases are detected early, and the cure rate is high [160, 161], but the prognosis of patients with recurrent endometrial cancer is still poor, so it is still necessary to find the best adjuvant treatment [162].

Ferroptosis inducers can also promote ferroptosis in endometrial cancer cells. The inducer Sulfasalazine can induce GSH consumption, ROS accumulation, and activation of C‐Jun N‐terminal kinases (JNK) in endometrial serous cancer cells. JNK is an effector of the RAS signaling pathway and activated JNK plays a synergistic role with accumulated ROS to induce ferroptosis in cancer cells [163]. Another study showed that *NSUN2* knockdown significantly increased the levels of lipid peroxides and lipid ROS in endometrial cancer cells, thereby enhancing the sensitivity of endometrial cancer to ferroptosis [164]. Mechanistically, *NSUN2* stimulated the m^5^C modification behavior of *SLC7A11* mRNA. As a reader of m^5^C, YBX1 directly recognizes and binds to m^5^C bits on *SLC7A11* mRNA through its internal cold shock domain (CSD). As a result, the stability of *SLC7A11* mRNA and the levels of SLC7A11 were increased. These results suggest that targeting the NSUN2/SLC7A11 axis inhibits tumor growth by increasing lipid peroxidation and ferroptosis of endometrial cancer cells in vitro and in vivo. In the estrogen‐induced mouse endometrial hyperplasia model, Cassia poria induces ferroptosis by inhibiting the p62/Kelch‐like epichlorohydrin‐associated protein 1 (Keap1)/Nrf2 signaling pathway [165], thus inhibiting endometrial hyperplasia. In addition, Cardanone in pecan peel is a novel ferroptosis inducer, which triggers ferroptosis in the endometrial cancer cell line Ishikawa through iron overload, GSH depletion, and lipid peroxidation [166]. The above studies show that RAS and Nrf2 signaling pathways regulate cell ferroptosis in endometrial cancer or endometrial hyperplasia, and the role of other related signaling pathways deserves further exploration.

Ferroptosis‐related genes are associated with dysregulation of immune cell infiltration in endometrial cancer. It is well known that the imbalance of immune cells in the tumor microenvironment promotes the growth of cancer cells, protects them from the attack of cytotoxic substances, promotes the proliferation of lymphatic endothelial cells and the formation of lymphatic vessels, and accelerates the metastasis of tumors. By analyzing 552 samples of endometrial cancer, Wei et al. screened out eight genes related to ferroptosis (*ACO1, ATP5MC3, GPX4, MDM2, PRKAA2, PHKG2, PRNP*, and *SLC11A2* genes) [167]. These eight genes are closely related to the infiltration levels of immune cells such as macrophages, neutrophils, B lymphocytes, CD8^+^T lymphocytes, and CD4^+^T lymphocytes [167]. According to the median risk score, endometrial cancer samples are divided into a low‐risk group and a high‐risk group, and it is found that the low‐risk group had a stronger immune killing ability of tumor cells, while the high‐risk group had stronger suppression of immune response. Liu et al.’s results show that in the gene expression profile of 511 cases of endometrial cancer patients, combined with clinical data, ferroptosis‐related gene expression is found to be positively correlated with immune checkpoint regulator CD40, programmed cell death ligand 1 (PD‐L1), and PD‐L2 [168]. These immune checkpoint‐related proteins are differentially expressed between the high‐risk and low‐risk groups. The response of patients in the low‐risk group to immunotherapy is significantly better than that in the high‐risk group. Yan et al.’s research viewpoint is that the overexpression of microsomal glutathione S‐transferase 1 in endometrial cancer can lead to a decrease in the infiltration levels of natural killer cells and CD8^+^T lymphocytes that mediate cytotoxic immune response [169]. Then, the number of myeloid suppressor cells that promote immune escape of tumor cells increases, leading to tumor progression and poor prognosis in patients with endometrial cancer. These results suggest that immune‐related ferroptosis genes can be promisingly used as markers to predict the response of patients to immunotherapy, and targeting these genes in combination with immunotherapy in the future may have better clinical effects.

Ferroptosis‐related genes can also be used as indicators to evaluate the prognosis of patients with endometrial cancer. A study reported that high expression of ferroptosis regulatory genes *CDKN1A, SLC7A11, SAT1*, and low expression of *ATP5MC3* can prolong the disease‐free survival time of patients with endometrial cancer [170]. These regulatory genes are also associated with tumor‐node‐metastasis stage, pathological grade, and overall survival time of endometrial cancer. According to 60 ferroptosis‐related genes and 544 endometrial cancer samples, 13 ferroptosis marker genes (*C11orf63, TUBB4A, LINC01224, KCNK6, TMPRSS2, SLC25A35, CD7, ZG16B, STX18, COL23A1, NWD1, GZMM*, and *NMU* genes) have been screened out [171], which are strongly associated with the disease development of endometrial cancer. The ferroptosis score calculated by principal component analysis can not only reflect the immune status and carcinogenic status of patients but also predict the prognosis of patients. Therefore, these genes can be evaluated in combination with FIGO staging in clinical practice to obtain better predictive value.

**FIGURE 4:**
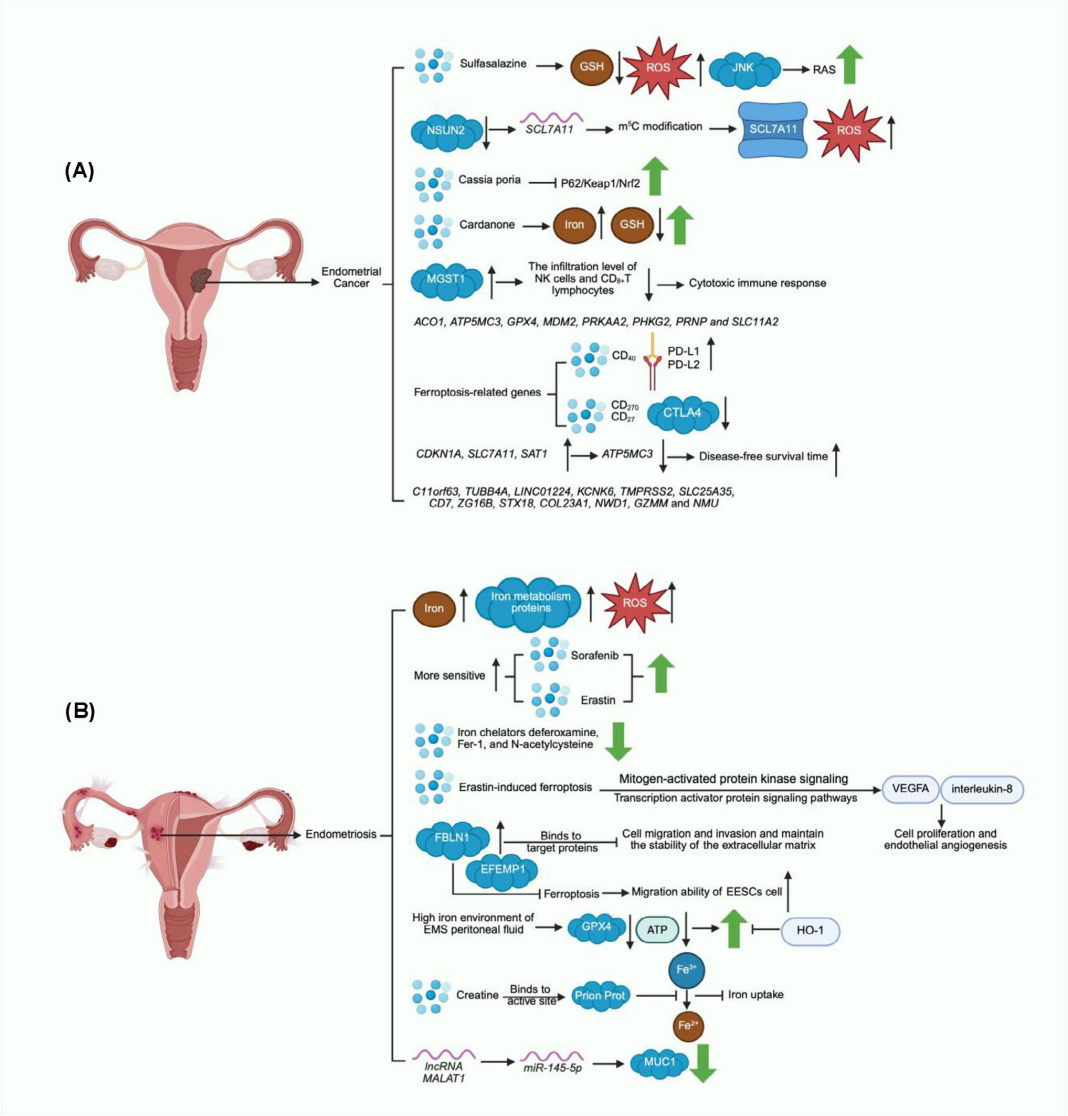
A graph of female reproductive system diseases. The abnormal endometrial tissue in (A) is endometrial cancer tissue, which mainly depicts the relationship between endometrial cancer and ferroptosis. The abnormal tissue of the endometrium in (B) is the endometriosis tissue, which mainly depicts the relationship between endometriosis and ferroptosis.

In conclusion, the in vitro research on the relationship between ferroptosis and endometrial cancer is not sufficient, and there is still a lot of research space worth exploring. Ferroptosis‐related genes are associated with the clinical outcome of patients with endometrial cancer and can be used as prognostic indicators (Figure 4A). Some of these prognostic genes are associated with dysregulation of tumor immune cell infiltration. Therefore, the regulation of these genes to restore dysfunctional immune cell infiltration may have greater clinical value in the future. In addition, there are currently some drugs on the market that act as ferroptosis inducers (such as Sulfasalazine, a clinically used drug for the treatment of chronic inflammatory diseases, which can target SLC7A11 to induce ferroptosis in cancer cells). In the future, more clinical multicenter drug trials for these drugs are needed to confirm the efficacy and safety of the drugs and reduce the harm of endometrial cancer to patients by using them alone or in combination.

### 4.4 Ferroptosis and Endometriosis

Endometriosis (EMS) is a disease in which endometrial glands and interstitial tissue are present outside the endometrium (e.g., ovaries, fallopian tubes, and abdominal cavity) [172]. Ischemia/reperfusion injury caused by retrograde menstrual blood induces ROS levels and local iron overload in focal areas, which is associated with increased sensitivity to local oxidative stress, immune inflammation, and ferroptosis in EMS [173, 174, 175, 176].

It has been reported that the ectopic endometrial tissue and peritoneal fluid of EMS patients are accompanied by increased expression of iron and iron metabolism proteins, accumulation of ROS and lipid ROS levels [177, 178], and similar phenomena have also been observed in animal models [179]. Ectopic endometrial stromal cells are more sensitive to the Sorafenib‐ and Erastin‐induced ferroptosis [178], and iron chelators deferoxamine, Fer‐1, and N‐acetylcysteine can reduce iron overload and inhibit inflammation and proliferation of stromal cells in animal models of ectopic endometrial tissue [140, 179]. Ferroptosis may be involved in the occurrence and development of EMS by affecting the invasion and migration, angiogenesis, and inflammatory stress of ESCs. Li et al. propose that the Erastin‐induced ferroptosis of embryonic stem cells (ESCs) may promote the expression of vascular endothelial growth factor A and interleukin‐8 through MAPK and transcription activator protein signaling pathways, thereby triggering cytokine secretion, promoting cell proliferation and endothelial angiogenesis, leading to the development of EMS [179]. Fibulin 1 (FBLN1) is a secreted glycoprotein that binds to target proteins to inhibit cell migration and invasion and maintain the stability of the extracellular matrix. According to the research results, the expression of FBLN1 in ectopic endometrial tissues is significantly increased, and it can be controlled by epidermal growth factor (EGF)‐containing fibulin‐like extracellular matrix protein 1 (EFEMP1), which inhibits ferroptosis process to mediate the increased migration ability of EESCs [180], suggesting the important role of FBLN1 in regulating the EFEMP1‐dependent ferroptosis pathway in the pathogenesis of EMS. In addition, it can be observed that the high iron environment of EMS peritoneal fluid can induce ferroptosis caused by GPX4 inactivation to affect blastocyst formation and lead to abnormal early embryo development [177]. On the other hand, local iron overload can lead to reduced Adenosine triphosphate (ATP) levels of embryos and hyperpolarization of mitochondrial membrane potential. Inhibition of HO‐1 plays an antiferroptosis role and improves mitochondrial damage and lipid oxidative accumulation in mouse embryos. At present, ferroptosis‐related genes have shown a good ability to discriminate and diagnose EMS [181, 182], and small molecular compounds such as Erastin and Fer‐1 can regulate the sensitivity of stromal cells to ferroptosis in the context of local iron overload, providing a new therapeutic strategy for the intervention of EMS. In the study by Chen et al. [183], it was observed that creatine binds to the active site of prion protein, inhibits the conversion of trivalent iron to divalent iron, reduces iron uptake, and promotes the tolerance of ectopic endometrial stromal cells to ferroptosis, contributing to the development of endometriosis. It has also been reported that *lncRN*A metastasis‐associated lung adenocarcinoma transcript 1 (MALAT1) acts as a competitive endogenous RNA of miR‐145‐5p to regulate the expression of ferroptosis inhibitor mucin 1 (MUC1) and promote erastin‐induced ferroptosis in endometriosis [184].

In conclusion, the occurrence of ferroptosis in EMS is closely related to ferroptosis (Figure 4B). Regulation of ferroptosis‐related targets may provide an enlightening therapeutic strategy for treating EMS in the future.

### 4.5 Ferroptosis and Polycystic Ovary Syndrome

Polycystic ovary syndrome (PCOS) is a common endocrine and metabolic disorder that affects women of reproductive age [185, 186, 187]. Its main manifestations are increased androgen secretion, polycystic ovary changes, and abnormal ovulation [188]. Increased iron storage in PCOS patients may lead to increased luteinizing hormone sensitivity and insulin resistance of ovarian cells, affecting the normal ovulation function and embryo implantation process of PCOS [189].

Different degrees of ferroptosis have been observed in PCOS patients and animal models [190]. Ferroptosis impairs the proliferation and secretion of granulosa cells and participates in the dynamic regulation process of PCOS pathophysiology. It is reported that *circRHBG* negatively regulates granulosa cell proliferation, and as a molecular sponge of *miR‐515‐5p*, it is involved in regulating SLC7A11 function and intracellular GSH/GSSG ratio and inhibits ferroptosis of GCs through System Xc and GPX4 pathways [191]. The biosynthesis of steroid hormones and hormone receptor response may involve genes related to ferroptosis, thus causing the disease development of PCOS [192]. In the study by Tan et al. [193], *miR‐93‐5p* expression was found to be upregulated in the granulation cells of patients with PCOS. Subsequent biological analysis and experiments showed that miR‐93‐5p can promote apoptosis and ferroptosis in granulation cells by regulating the NF‐κB signaling pathway. In addition, homocysteine is associated with insulin resistance and disrupted sex hormone levels and may be an important trigger for PCOS. Homocysteine can promote the methylation of GPX4, which triggers ferroptosis and oxidative stress [194]. In the study by Shi et al. [195], Fer‐1, as a ferroptosis inhibitor, can promote the demethylation process by regulating the activity of methylase, so as to prevent the damage of ovarian granulosa cells mediated by homocysteins and thus improve the symptoms of PCOS. Mobilization of free iron pool and intracellular GSH cycling plays a key regulatory role in ferroptosis [196, 197], and antioxidant factors such as NRF2 and SOD1 are associated with fetal loss in PCOS pregnant rats [198]. However, the expression of some ferroptosis‐related genes, such as dipeptidyl peptidase 4 and CDGSH iron‐sulfur domain 1, is not consistent in the uterus and placenta. Significant changes in the expression of necrotic apoptosis of the uterus and regulatory molecules related to apoptosis pathways can be observed in PCOS rat pregnancy models [199], suggesting that multiple programmed cell death modes may interact and be related to the oxidative stress process of PCOS. It has also been reported that the application of antiandrogens and antioxidants can improve the sensitivity to ferroptosis in the pathological state of PCOS, which is manifested by the upregulated expression of core proteins SLC7A11, GSH, and GPX4, and the recovery of iron deposition, antioxidant capacity, and lipid peroxidation levels [196, 200]. In the report by Yang et al. [195], Nuciferine was found to protect ovarian granulosa cells from hyperandrogenism by inhibiting ferroptosis through SOX2‐mediated activation of the SLC7A11/GPX4 axis [201].

**FIGURE 5:**
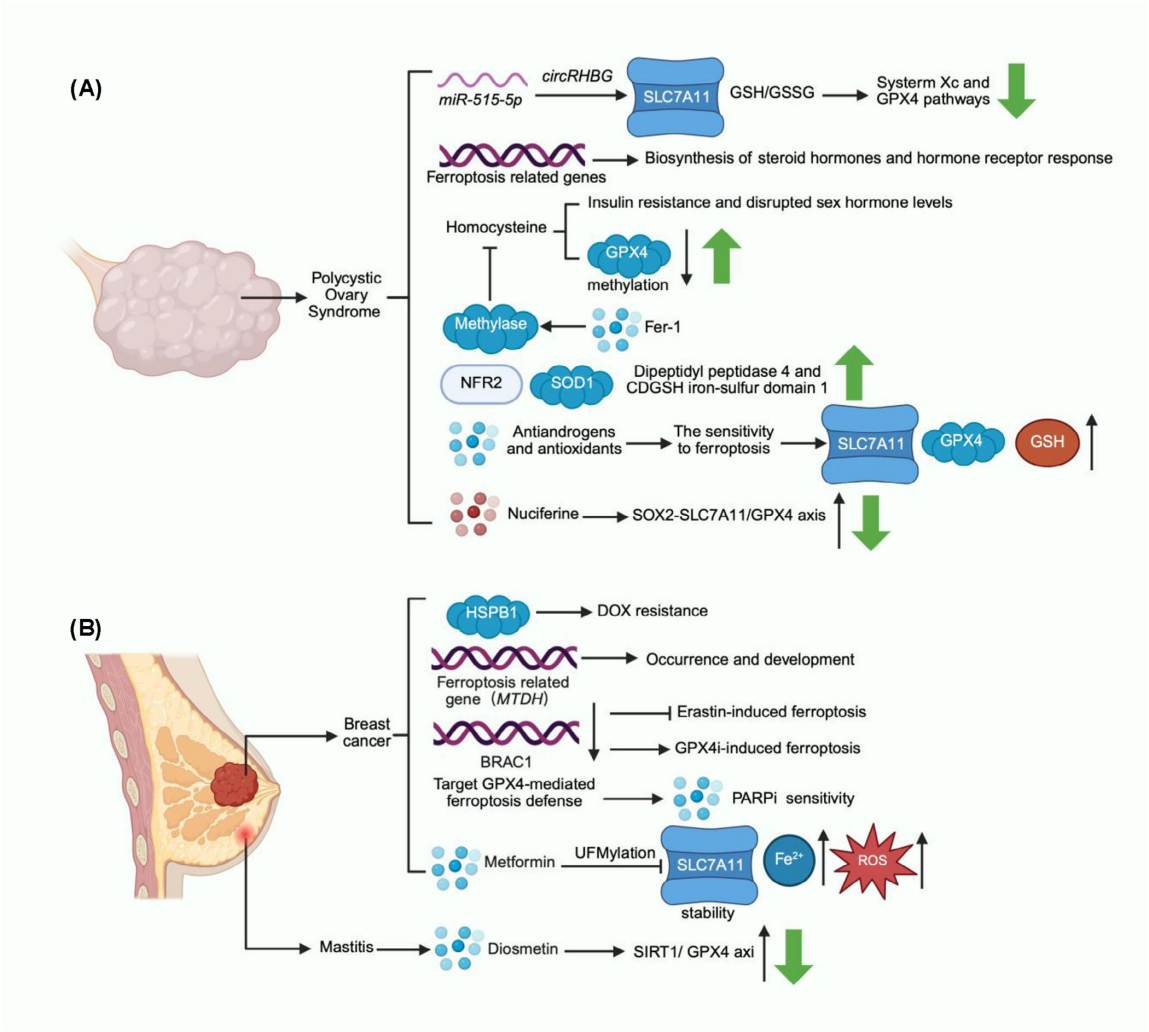
A graph of female reproductive system diseases. The polycystic ovarian change in (A) is polycystic ovary syndrome, which mainly depicts the relationship between polycystic ovary syndrome and ferroptosis. The abnormal breast tissue in (B) is breast cancer and mastitis. The relationship between breast‐related diseases and ferroptosis is described.

In conclusion, the occurrence of PCOS is closely related to ferroptosis (Figure 5A), and the induction of ferroptosis may offer a new therapeutic strategy for ovulation management in patients with polycystic ovary syndrome.

All the above reports suggest that ferroptosis is correlated with the onset and development of common gynecological diseases. Considering the association between ferroptosis and pregnancy‐related diseases, it can be found that the female reproductive system is closely related to ferroptosis. In addition, we have collated animal experiments on the mechanisms of ferroptosis in the female reproductive system, further supporting the correlation between ferroptosis and the female reproductive system (Table 1). In the future, ferroptosis therapy may become an effective direction for early screening and treatment of female reproductive system‐related diseases.

**TABLE 1:** Animal experiments on ferroptosis in female diseases.

Animal experiments	Pathway/Mechanism	Ferroptosis levels	Reference
GDM mice	1. Adiponectin inhibits the blood sugar and lipid levels, restores CPT‐1	↓	[66]
PE rat	1. *miR‐30b‐5p* downregulating or Fer‐1 blocking ferroptosis phenotype	↑	[81, 82]
Preterm birth mice	1. *PLA2G6* metabolize Hp‐PE to oxidized fatty acids and alleviate GPX4	↓	[52]
Miscarriage mice	1. Upregulation of HIF1α‐SUMO‐mediated transcription of *NCOA4*.	↑	[101]
Ovarian cancer mice	1. Combination of Sulfasalazine, Olaparib, prolong the survival time	↑	[132]
Cervical cancer mice	1. MTCH1 combined with Sorafenib, inhibited cervical cancer growth	↑	[142]
Endometrial hyperplasia mouse model	1. Cassia poria inhibiting Keap1/Nrf2 signaling pathway thus inhibiting endometrial hyperplasia.	↑	[165]
Ectopic endometrial animal model	1. Fer‐1, and N‐acetylcysteine can reduce iron overload 2. Inhibition of HO‐1 can play an antiferroptosis role and improve mitochondrial damage and lipid oxidative accumulation	↓	[177, 179]
PCOS rat pregnancy model	1. NRF2 and SOD1 are associated with fetal loss in PCOS	↑	[198]

## 5 Ferroptosis and Breast‐Related Diseases

BC is a common cancer in women [202, 203, 204, 205]. Although the breasts do not belong to the female reproductive system, as a disease with a high mortality rate affecting female health, the role of ferroptosis in BC is also worthy of our attention. Globally, the number of newly diagnosed cases of female BC remains high, with the incidence and mortality of female BC in 2018 being 24.2% and 15%, respectively [206]. Therefore, we still need to find a better solution.

At present, drug resistance of BC is the bottleneck of clinical efficacy. Overcoming drug resistance in tumors is an urgent problem. Ferroptosis has been found to play a vital role in BC resistance. In the view of Liang et al. [207], a new functional role for heat shock protein beta‐1 (HSPB1) is to regulate chemotherapy resistance and ferroptosis in BC cells. HSPB1 promotes doxorubicin (DOX) resistance by protecting BC cells from drug‐induced ferroptosis. As reported by Shen et al. [208], the DNAJC12‐HSP70‐AKT signaling axis mediates chemotherapy resistance in BC by inhibiting the DOX‐induced ferroptosis and apoptosis. However, in the report of Hu et al. [209], the ferroptosis‐related gene *MTDH* regulates the occurrence and development of BC. In addition, Lei et al.’s study revealed for the first time that *breast cancer susceptibility gene 1 (BRCA1)*‐deficient cancer cells were resistant to Erastin‐induced ferroptosis, but showed vulnerability to GPX4 inhibitors (GPX4i)‐induced ferroptosis [210]. A novel therapeutic strategy for *BRCA1*‐deficient tumors is proposed: targeting GPX4‐mediated ferroptosis defense to enhance the sensitivity of Resistance to poly (ADP‐ribose) polymerase inhibitors (PARPi) and overcome tumor resistance to PARPi. Studies have shown that metformin reduces the protein stability of SLC7A11 by inhibiting UFMylation, thereby increasing intracellular Fe^2+^ and lipid ROS levels. At the same time, metformin is also used in combination with Salazepyridine (systemic XC‐inhibitor) to synergically induce ferroptosis and inhibit the proliferation of BC cells [211]. In addition, diosmetin has been shown to inhibit *Staphylococcus aureus*‐induced mastitis by inhibiting SIRT1/GPX4‐mediated ferroptosis and reducing inflammation [212].

All the above studies indicate the correlation between ferroptosis and breast‐related diseases (BC, mastitis, *etc*.), which provides a new direction for the further treatment of breast‐related diseases. For the convenience of reading, we have summarized the changing trends of ferroptosis‐related markers and genes in female health and diseases mentioned above (Table 2).

**TABLE 2:** Trends in ferroptosis markers and genes in female health and disease.

Disease	Ferroptosis‐related marker/gene	Ferroptosis levels change mechanism	Reference
GDM	GPX4 ↓	High‐glucose leads lipid peroxidation activated, ROS and MDA concentrations increased	[64]
PE	FTH1, FTL, FPN1, SLC7A11↓ GPX4, NRF2 ↑	Placental regional dysplasia and hypoxic‐ischemic environment leads ROS accumulation, oxidative stress increased, dysregulated iron and lipid metabolism	[72]
Preterm birth	GPX4 ↓ *PLA2G6* ↑	Placental lipid metabolome dysregulation, including increased Hp‐PE and 4‐HNE	[64, 96, 97]
Miscarriage	TfR1, ACSL4, SOD, NCOA4↑		
POI	GPX4 ↓	*BNC1* deficient triggers oocyte ferroptosis through the NF2‐YAP pathway	[108]
Ovarian cancer	ACSL4, COX‐2, MCU ↓ GPX4, FTH1, NCOA4 ↑ *DNAJB6, BACH1, ALOX12* ↑ *RB1 hsa_circ_0001546* ↓	Cytoplasmic oxidative stress and mitochondrial dysfunction exist in ovarian cancer cells, and the high level of ROS leads to the occurrence of ferroptosis	[135]
Cervical cancer	MTCH 1, GPX4 ↓ ACSL4 ↓ NRF2, GPX4, SLC7A11↑	Mitochondrial oxidative phosphorylation is disrupted and ROS increased by decreasing NAD^+^ levels, which causes GSH, GPX4 inactivation, and lipid peroxide accumulation	[142].
Endometrial cancer	SLC7A11, Nrf2 ↑ *CDKN1A, SLC7A11, SAT1* ↑ *ATP5MC3* ↓	Elevated lipid peroxides and lipid ROS in endometrial cancer induce ferroptosis	[170].
Endometriosis	FBLN1↑ GPX4, HO‐1, MUC1↓	Ischemia/ reperfusion injury caused by retrograde menstrual blood induces ROS levels and local iron overload in focal areas.	[173, 174, 175, 176].
PCOS	SLC7A11, GPX4 ↓ NRF2, SOD1 ↑ *miR‐515‐5p, miR‐93‐5p* ↑	miR‐93‐5p promotes apoptosis and ferroptosis in granulation cells by regulating the NF‐κB signaling pathway	[193].
BC	SLC7A11, GPX4 ↓ *MTDH* ↑	BC induces ferroptosis by increasing intracellular Fe^2+^ and lipid ROS levels	[209]

## 6 Ferroptosis and Other Related Diseases

In addition to the diseases described above, there are a number of female diseases that are also strongly associated with ferroptosis (Table 2). Recent evidence suggests that the emerging role of iron overload and ferroptosis in female infertility is to induce hypogonadism, resulting in ovarian dysfunction, damage to preimplantation embryos, diminished endometrial receptivity, and crosstalk between subfertility‐related disorders such as polycystic ovary syndrome and endometriosis [213]. In the report of Fan et al., it was pointed out that tubal factor infertility is characterized by ectopic pregnancy caused by tubal obstruction, which is manifested in the accelerated accumulation of lipids and ROS [197], consistent with the trend of ferroptosis [4]. In addition, the gut microbiota and its metabolites are involved in iron metabolism, ferroptosis, and female infertility. Furthermore, it shows that the relationship between ferroptosis and female infertility is close. In another study [214], investigators demonstrated an increased ferroptosis burden in the endometrium of Intrauterine adhesions (IUA). Moreover, Erastin‐induced ferroptosis promoted EMT and fibrosis of endometrial epithelial cells in vitro. In parallel, Fer‐1 significantly ameliorated endometrial fibrosis in a double‐injury IUA mouse model. It is demonstrated that increased ferroptosis leads to fibrosis of the endometrial adhesions in utero. Sun et al. found that exposure to bisphenol A (BPA) during pregnancy causes fetal growth restriction [215]. It was subsequently confirmed in their study that *YAP* or *TAZ* siRNA enhanced BPA‐induced ferroptosis, suggesting that trophoblast siderosis depends on *YAP/TAZ* downregulation after BPA stimulation. It has also been shown that caffeic acid inhibits inflammation and siderosis by regulating the AMPKα/mTOR/HIF‐1α signaling pathway to alleviate *Staphylococcus aureus*‐induced endometritis [216]. In addition, SmgGDS (small GDP‐binding protein dissociative stimulators) have been shown to modulate estradiol‐dependent cardioprotective effects via the AMPK/mTOR signaling pathway, inducing the occurrence of takotsubo syndrome [217].

These diseases fully demonstrate the close relationship between ferroptosis and a variety of women's health and diseases (Table 3). Ferroptosis, as one of the nonprogrammed cell death behaviors, may play an important role as a method of disease diagnosis and treatment.

**TABLE 3:** Ferroptosis in other female‐related diseases.

Disease	Mechanisms	Ferroptosis levels	Reference
Infertility	Iron overload and ferroptosis can induce hypogonadism, lead to ovarian dysfunction, damage preimplantation embryos, and attenuate endometrial receptivity. Tubal factor infertility accelerates accumulation of lipids and ROS; The gut microbiota and its metabolites are involved in iron metabolism, ferroptosis, and female infertility.	↑	[213]
Intrauterine adhesions	1. An increased ferroptosis burden in the endometrium of Intrauterine adhesions; 1. Erastin‐induced ferroptosis promoted EMT and fibrosis of endometrial epithelial cells in vitro; 2. Fer‐1 significantly ameliorated endometrial fibrosis in a double‐injury IUA mouse model.	↑	[214]
Fetal growth restriction	Exposure to bisphenol A (BPA) during pregnancy causes fetal growth restriction; YAP or TAZ siRNA enhanced BPA‐induced ferroptosis	↑	[215]
Endometritis	Caffeic acid inhibits inflammation and siderosis by regulating the AMPKα/mTOR/HIF‐1α signaling pathway.	↓	[216]
Takotsubo syndrome	SmgGDS modulates estradiol‐dependent cardioprotective effects via the AMPK/mTOR signaling pathway	↓	[217]

## 7 Therapeutic Advances Targeting Ferroptosis

For the convenience of reference, we have summarized some current drugs for the targeted treatment of ferroptosis. From the three pathways of coordinated control of ferroptosis by iron metabolism, redox, and lipid metabolism, respectively, the small molecule inducers or inhibitors and natural compounds that affect the mechanism of ferroptosis are listed [218].

### 7.1 Small Molecule Inhibitors/Inducers of Ferroptosis

The main small‐molecule compounds mediating the iron metabolism pathway in ferroptosis include various inhibitors (Compound 9a, ciclopirox, deferoxamine, deferiprone, deferasirox, and dexrazoxane) and inducer JQ1 [219, 220, 221, 222]. There are currently no inducers or inhibitors for female disease models in the iron metabolism pathway. The main compounds in the redox pathway of ferroptosis include multiple inhibitors (aminooxyacetic acid, curculigoside, dihydroartemisinin, Edaravone, Fer‐1, Liproxsatin‐1, NAC, and UAMC‐3203) and multiple inducers (acetaminophen, auranofin, Brequinar, buthionine sulphoximine, CH004, Erastin, Erastin‐acetaminophen, imidazole ketone erastin, piperazine erastin, RSL3, sulfasalazine, TRG, TRG+erastin+Sorafenib, and WA) [8, 34, 222, 223, 224, 225, 226, 227, 228]. Among them, Sulfasalazine and Olaparib were applied in the ovarian cancer disease model [229, 230]. Sorafenib and NAC were, respectively, utilized in disease models of cervical cancer and endometriosis. Ferrostatin‐1 and Erastin have been involved in multiple studies on female diseases. The main compounds in the lipid metabolism pathway of ferroptosis include various inhibitors (Baicalein, IMA‐1, ML355, NDGA, PRGL493, Zileuton) and various inducers (Pioglitazone, Rosiglitazone, and Troglitazone) [229, 231, 232, 233]. Among them, Pioglitazone has been applied for treating BC. Therefore, there are still a large number of compounds that have not been applied to cure female diseases, and the possibilities need to be continuously explored in the future.

### 7.2 Natural Compounds Modulating Ferroptosis

In addition to small molecule inhibitors, some natural compounds can effectively regulate ferroptosis. Among them, some iron chelators, Glutathione, Selenium, and RTAs can effectively inhibit ferroptosis, whereas compounds such as Trigonelline effectively stimulate ferroptosis [223].

Among the natural compounds described above, there are those with relatively clear pharmacological mechanisms studied. However, in this article, we have also summarized that some natural compounds play a certain role in female health and diseases, such as melatonin and adiponectin, that can protect patients with GDM by inhibiting ferroptosis. MAP30 and artesunate can induce the occurrence of ferroptosis and thereby inhibit the occurrence and progression of ovarian cancer. Oleanolic acid, triptolide, Cassia poria, and Cardanone can induce the occurrence of ferroptosis and protect patients with cervical cancer from invasion. Nuciferine inhibits ferroptosis to protect PCOS patients, while caffeic acid can inhibit ferroptosis and thus prevent damage from endometritis. At present, the mechanism of action of these natural compounds remains unclear, and further in‐depth research on the mechanism is needed.

### 7.3 Ferroptosis‐Based Combination Therapies

At present, ferroptosis, as a nonapoptotic programmed cell death, is mainly used as an emerging therapeutic target for cancer treatment. However, with the continuous development of biotechnology in the field of biology, there are currently ferroptosis‐driven nanotherapeutics for combined treatment. During this process, ferroptosis is combined with other different therapeutic strategies such as tumor imaging, immune regulation, chemotherapy, and phototherapy [234], which is highly expected in future treatments and is one of the possible directions for the treatment of female tumors.

### 7.4 Current Challenges and Clinical Trials

At present, most of the studies on the mechanism of ferroptosis are still at the level of animal models and/or cell models, and there are a large number of challenges that prevent the possibility of further clinical practice. Although many molecules have been proven to regulate ferroptosis, the drug‐available molecules for humans are very limited. Moreover, poor pharmacokinetic problems also limit the further development of drugs targeting ferroptosis. We have discussed herein some issues regarding the drug resistance mechanism of gynecological tumors. Ferroptosis, as a means of synergistic treatment, has great research space and development value. Therefore, some researchers are exploring the possibility of some traditional herbs targeting and regulating ferroptosis, hoping to develop more effective drugs for targeted ferroptosis treatment

## 8 Conclusion and Perspectives

With the continuous in‐depth study of ferroptosis, it is closely related to the life and health of women. The research on common pregnancy diseases, gynecological diseases, and malignant tumors is the focus of our attention. In this regard, from the perspective of disease development, the molecular mechanism of ferroptosis regulating disease development in women is expounded.

According to the content of the summary, there are several research directions worthy of further exploration. First, ferroptosis is regulated by a complex network composed of genetic, immune, metabolic, and other mechanisms. However, the regulatory mechanisms between ferroptosis and different female diseases have not yet been thoroughly studied. It is still difficult to clarify the relationship between ferroptosis‐related targets and specific signaling pathways and female diseases, and it is difficult to conduct accurate treatment. Therefore, continuous in‐depth exploration is needed. Second, some drugs currently on the market may have mechanisms to induce or inhibit ferroptosis in female diseases but lack theoretical support, which deserves further investigation. Launching more sustained basic and clinical drug trials and translating these drugs into clinical treatments earlier could provide new ways to address clinical treatment issues. Finally, most of the current research on ferroptosis between the above diseases and other disciplines mainly focuses on the analysis of biogenesis and the study of drug resistance mechanisms. Its detection methods are relatively limited, such as immunohistochemistry of pathological tissue sections. However, it is not possible to perform continuous invasive procedures to obtain specimens during clinical treatment to verify the therapeutic effect of ferroptosis. Therefore, it is still necessary to find a simpler and more standardized detection means to better detect the occurrence of ferroptosis and thereby intervene early.

Ferroptosis plays an important role in the occurrence and development of female diseases. There is a strong relationship between ferroptosis and common pregnancy diseases. It is believed that simple and convenient detection methods will be found in the future so that their related genes can be used as early screening indicators for common obstetric diseases. In addition, ferroptosis‐related genes are used as prognostic indicators in gynecological malignant tumors and are even closely related to tumor immune cell infiltration disorder and tumor drug resistance. Therefore, in the future, targeted ferroptosis therapy for common pregnancy diseases, combined with conventional chemoradiotherapy and immunotherapy for gynecological malignant tumors, may become an emerging treatment strategy to reduce the mortality of certain diseases.

## Author Contributions

Qiang Xu, Chongying Zhu, and Lin Li wrote the original manuscript. Qiang Xu, Jiayong Li, and Zihao An prepared the figures and tables. Chao Tang designed and edited the manuscript. All authors have read and approved the final manuscript.

## Conflicts of Interest

The authors declare no conflicts of interest.

## Ethics Approval

Not applicable.

## Data Availability

All data generated or analyzed during this study are included in this published article.
